# Differentially Expressed Genes Related to Isoflavone Biosynthesis in a Soybean Mutant Revealed by a Comparative Transcriptomic Analysis

**DOI:** 10.3390/plants13050584

**Published:** 2024-02-21

**Authors:** Jung Min Kim, Jeong Woo Lee, Ji Su Seo, Bo-Keun Ha, Soon-Jae Kwon

**Affiliations:** 1 Advanced Radiation Technology Institute, Korea Atomic Energy Research Institute, Jeongeup 56212, Republic of Korea; jmkim0803@kaeri.re.kr (J.M.K.); jeongwoolee9529@kaeri.re.kr (J.W.L.); su1545@kaeri.re.kr (J.S.S.); 2 Department of Applied Plant Science, College of Agriculture and Life Sciences, Chonnam National University, Gwangju 61186, Republic of Korea

**Keywords:** soybean, isoflavone, RNA-seq, differentially expressed genes, gene ontology, Kyoto Encyclopedia of Genes and Genomes

## Abstract

Soybean [*Glycine max* (L.) Merr.] isoflavones, which are secondary metabolites with various functions, are included in food, cosmetics, and medicine. However, the molecular mechanisms regulating the glycosylation and malonylation of isoflavone glycoconjugates remain unclear. In this study, we conducted an RNA-seq analysis to compare soybean genotypes with different isoflavone contents, including Danbaek and Hwanggeum (low-isoflavone cultivars) as well as DB-088 (high-isoflavone mutant). The transcriptome analysis yielded over 278 million clean reads, representing 39,156 transcripts. The analysis of differentially expressed genes (DEGs) detected 2654 up-regulated and 1805 down-regulated genes between the low- and high-isoflavone genotypes. The putative functions of these 4459 DEGs were annotated on the basis of GO and KEGG pathway enrichment analyses. These DEGs were further analyzed to compare the expression patterns of the genes involved in the biosynthesis of secondary metabolites and the genes encoding transcription factors. The examination of the relative expression levels of 70 isoflavone biosynthetic genes revealed the *HID*, *IFS*, *UGT*, and *MAT* expression levels were significantly up/down-regulated depending on the genotype and seed developmental stage. These expression patterns were confirmed by quantitative real-time PCR. Moreover, a gene co-expression analysis detected potential protein–protein interactions, suggestive of common functions. The study findings provide valuable insights into the structural genes responsible for isoflavone biosynthesis and accumulation in soybean seeds.

## 1. Introduction

In plants, phytochemicals are naturally produced primary and secondary metabolites with antioxidant and antimicrobial properties. They protect against fungi, bacteria, and viruses, while also deterring herbivorous insects and animals [1]. Isoflavones, which are synthesized exclusively by leguminous crops, especially soybeans, are important for legume–microbe interactions and root nodulation [2]. Soybean isoflavones, which are phytoestrogens structurally similar to mammalian estrogen (17β-estradiol), have medicinal properties useful for treating osteoporosis, heart disease, hormone-related cancers, and menopausal symptoms [3,4]. The global consumption of soybeans has increased substantially because of their positive effects on human health, serving as natural sources of secondary metabolites, including isoflavones, tocopherols, and saponins at a low cost [5]. In the context of soybean breeding, manipulating isoflavone biosynthesis can create new soybean cultivars with optimal isoflavone contents. Despite extensive research on the mechanisms underlying the glycosylation and malonylation of isoflavone, the genetic basis of isoflavone accumulation remains unclear, possibly because of the challenges associated with identifying the key genes responsible for the production of isoflavone glycoconjugates. The soybean isoflavone content varies depending on developmental and environmental conditions. More specifically, the isoflavone content is a quantitative trait influenced by multiple genetic factors and external stimuli, including biotic and abiotic stresses [6,7,8].

In soybeans, the biosynthesis of isoflavones involves several enzyme-catalyzed reactions in a branch of the phenylpropanoid pathway. Phenylalanine is the initial substrate of the pathway, which generates isoflavones that are subsequently stored in vacuoles in the following four distinct forms: aglycones (daidzein, glycitein, and genistein), glycosides (daidzin, glycitin, and genistin), malonyl glycosides (6″-*O*-malonyl daidzin, 6″-*O*-malonyl glycitin, and 6″-*O*-malonyl genistin), and acetyl glycosides (6″-*O*-acetyl daidzin, 6″-*O*-acetyl glycitin, and 6″-*O*-acetyl genistin). Among these forms, glycosides and malonyl glycosides are particularly abundant in soybean seeds [9,10]. Isoflavone biosynthesis begins with phenylalanine ammonia-lyase (PAL) catalyzing the removal of an amine group to convert phenylalanine to cinnamic acid, which is then converted to p-coumaric acid by cinnamic acid 4-hydroxylase (C4H). The following step involves the conversion of p-coumaric acid to p-coumaroyl CoA by 4-coumarate:coenzyme A ligase (4CL). Chalcone synthase (CHS)/chalcone reductase (CHR) mediate the synthesis of chalcones, which are converted to isoliquiritigenin and naringenin by chalcone isomerase (CHI) [11]. Isoflavone synthase (IFS) competes with other endogenous flavonoid-related enzymes to convert naringenin into genistein, whereas 2-hydroxyisoflavanone dehydratase (HID) catalyzes the dehydration that results in the production of daidzein and genistein [12,13]. The *IFS* genes, which are homologous to genes in the cytochrome P450 monooxygenase family, include *IFS1* and *IFS2.* These crucial metabolic genes are responsible for synthesizing all isoflavonoids [12]. Subsequently, UDP-glycosyltransferases (UGTs) catalyze the 7-*O*-glycosylation that converts aglycones to glycosides [14]. Malonyl and acetyl glycosides are then synthesized via acylations involving malonyl-CoA:flavonoid acyltransferases (MaTs), including benzylalcohol *O*-acetyltransferase (BEAT), anthocyanin *O*-hydroxycinnamoyltransferase (AHCT), anthranilate N-hydroxycinnamoyl/benzoyltransferase (HCBT), and deacetylvindoline 4-*O*-acetyltransferase (DAT). This group of enzymes belongs to the BAHD acyltransferase family [15,16]. To date, more than 100 BAHD genes have been identified in legumes, including *GmIMaT1*, *GmMaT2*, *GmIMaT3*, and *GmMT7* in soybean [14,17,18]. The ATP-binding cassette (ABC) and multidrug and toxic compound extrusion (MATE) transporters contribute to the accumulation of isoflavone glycosides and their secretion mediated by vacuolar transporters. Biosynthetic genes, including *IFS*, *HID*, and *UGT*, are co-expressed with the genes encoding the ABC and MATE transporters for both isoflavone and saponin in soybean roots [19]. Despite the importance of the BAHD family, it has not been thoroughly characterized at the genetic level.

The malonylated isoflavones 6″-*O*-malonyl genistin and 6″-*O*-malonyl daidzin are major constituents in soybean seeds, whereas glycitein and its glycosides are present in smaller proportions [20]. The enzyme IF7MaT, which exhibits high specificity for isoflavone glycosides, catalyzes the malonylation of the corresponding precursors, thereby contributing to soybean isoflavone biosynthesis [21]. The overexpression of *GmIMaT1* and *GmIMaT3* results in substantial increases (50% to 100%) in malonyl daidzin and malonyl genistin contents in transgenic soybean hairy roots, whereas down-regulating the expression of these genes leads to decreased malonyl glycoside contents and changes to the total isoflavone (TI) content [17]. By manipulating gene expression in soybeans, Ahmad et al. [18] functionally characterized *MaT* genes. Specifically, the overexpression of *MaT* genes promotes soybean nodulation and secretion through isoflavone malonylation, whereas knocking down the expression of these genes leads to decreases in malonylated isoflavone levels in transgenic hairy roots. Glycosylation and malonylation are important for signaling in nitrogen-fixing nodules, but they also modulate solubility, stability, and transport and storage in vacuoles [22]. Transcription factors (TFs) from several families, including MYB, bZIP, WRKY, bHLH, MADS-box, and WD40, regulate the expression of the structural genes in the phenylpropanoid pathway in higher plants [23,24]. For example, the R3-type MYB TF GmMYB29 concurrently activates the *IFS2* and *CHS8* promotors; the overexpression of *GmMYB29* increases the isoflavone content in transgenic soybean hairy roots [25]. Silencing *OscWRKY1* down-regulates the expression of *PAL*, *C4H*, and *4CL* in the phenylpropanoid pathway, resulting in a decrease in the rosmarinic acid content and an increase in the susceptibility to bacterial pathogens [26]. In *Arabidopsis thaliana*, the WD40–bHLH–MYB complex reportedly regulates the co-expression of multiple genes, thereby controlling anthocyanin biosynthesis [27].

Mutation breeding, which is an efficient strategy for accelerating crop improvement, has been broadly adopted to quickly generate new cultivars and diverse populations of various species [28,29]. To date, more than 3402 varieties of approximately 210 crop species have been registered in the International Atomic Energy Agency/Mutant Variety Database (MVD). A total of 182 soybean mutants have been registered in MVD (https://www.iaea.org/) (accessed on 1 January 2024).

These mutant varieties incorporating functional genes are valuable materials for breeding and genetic analyses, including quantitative trait locus mapping and genome-wide association studies [30,31,32]. We previously developed a mutant diversity pool (MDP) comprising 203 soybean mutants for breeding as well as for marker selection, gene expression analyses, and genome-wide association studies of agronomic traits and functional components [33,34,35,36]. The MDP was also used to identify a new soybean mutant (DB-088), which was derived from wild-type Danbaek seeds that were irradiated (250 Gy ^60^Co gamma-irradiation). A 3-year comparison revealed the isoflavone content of DB-088 (6993–9975 µg g^−1^) was significantly higher than that of Danbaek (1543–2952 µg g^−1^) (Figure S1).

The objective of this study was to identify and characterize the main genes involved in isoflavone biosynthesis in soybean seeds. DB-088 has inferior agronomic traits and indeterminate growth, resulting in delayed flowering and maturation compared to wild-type Danbaek. In contrast, Hwanggeum has superior agronomic traits such as determinate growth, large seeds, early flowering, and early maturation. To challenge soybean cultivation and breeding with optimal isoflavone content, we performed a transcriptome sequencing (RNA-seq) analysis to reveal the differences in gene expression levels between DB-088 (high isoflavone content) and soybean cultivars Hwanggeum and Danbaek (low isoflavone contents), with a particular focus on the downstream regulatory genes in the isoflavone biosynthetic pathway. The study data will be useful for clarifying the genetic basis of isoflavone biosynthesis and for breeding novel soybean varieties with high isoflavone contents.

## 2. Results

### 2.1. Variations in Isoflavone Contents among Soybean Seed Developmental Stages

**Figure 1:**
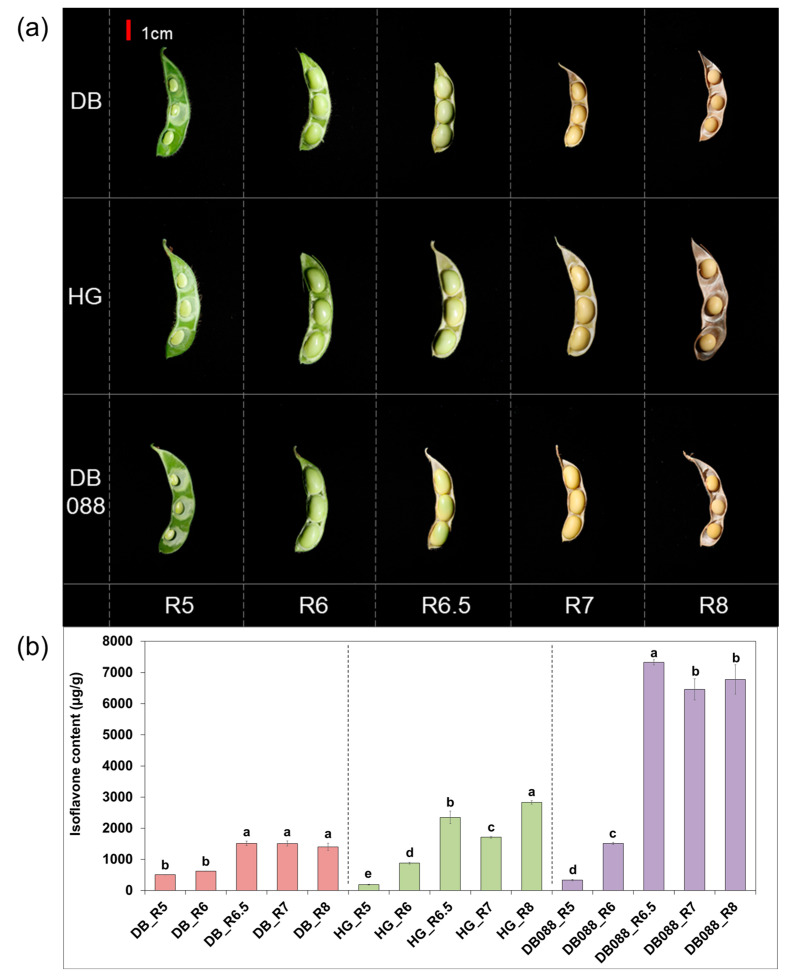
Total isoflavone content in different soybean seed developmental stages. DB, Danbaek; HG, Hwanggeum. (**a**) Representative soybean seed developmental stages. R5: 25 days after flowering (DAF), R6: 45 DAF, R6.5: 55 DAF, R7: 65 DAF, and R8: 80 DAF. (**b**) Total isoflavone content across seed developmental stages. Lowercase letters above the bars indicate significant differences between stages at the 5% level according to Fisher’s LSD test (*n* = 3).

The analysis of individual isoflavone and total isoflavone (TI) contents of Hwanggeum, Danbaek, and DB-088 seeds at different developmental stages revealed obvious differences in all isoflavones (Table S1 and Figure 1a,b). Hwanggeum and DB-88 seeds predominantly accumulated acetyl glycosides during the seed-filling stage (R5). After the R6 stage, malonyl glycosides constituted the largest proportion (more than 70%) of the TI content of all genotypes. Notably, the TI content increased rapidly by 2.43-, 2.67-, and 4.84-fold between R6 and R6.5. In the R6.5 stage, the isoflavone content of DB-088 (7321.88 µg g^−1^) was 4.84- and 3.12-fold higher than that of Danbaek (1512.82 µg g^−1^) and Hwanggeum (2347.78 µg g^−1^), respectively.

At full maturity, the TI content of DB-088 (6778.34 µg g^−1^) was 4.83- and 2.39-fold higher than that of Danbaek (1403.34 µg g^−1^) and Hwanggeum (2831.68 µg g^−1^), respectively. In particular, malonyl glycosides accounted for over 73% of the TI content. For all genotypes, there were significant differences in individual and TI contents during seed maturation, suggesting the expression of isoflavone biosynthetic genes is differentially regulated among genotypes and reproductive stages.

### 2.2. Transcriptome Sequencing Data Analysis

**Figure 2:**
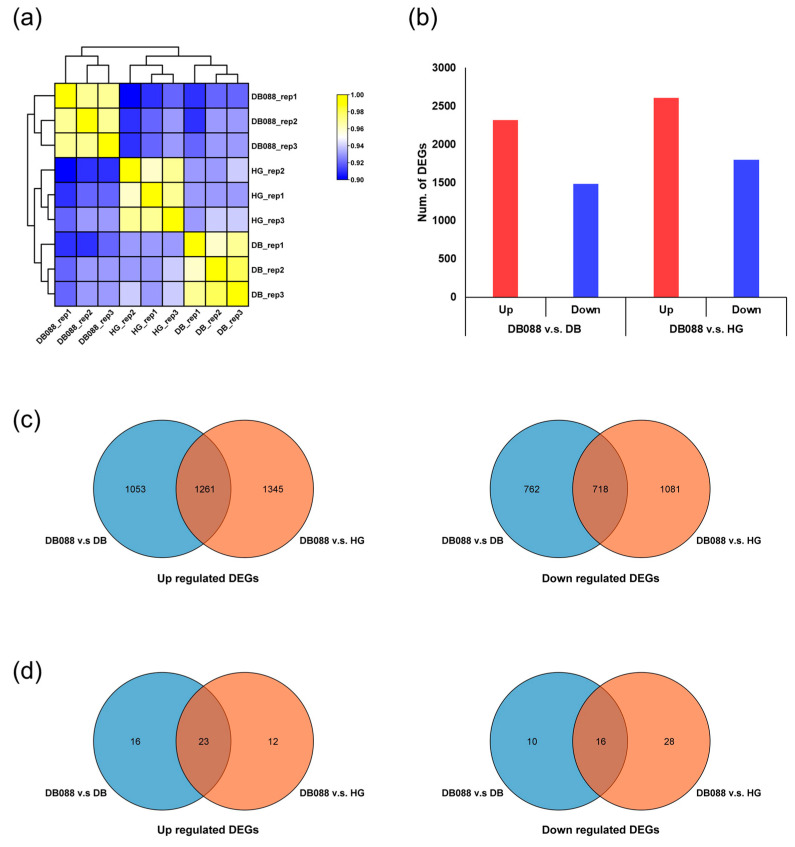
Comparative analysis of gene expression profiles and differential expression. (**a**) Hierarchical clustering illustrating the relationships between samples on the basis of Pearson correlation coefficients. (**b**) Comparison of the number of DEGs between DB-088 and Danbaek/Hwanggeum. (**c**) Venn diagram presenting the up/down-regulated DEGs among the DEGs revealed by the comparisons between DB-088 and Danbaek/Hwanggeum. (**d**) Venn diagram presenting the up/down-regulated isoflavone biosynthetic DEGs revealed by the comparisons between DB-088 and Danbaek/Hwanggeum.

The RNA-seq analysis involved three replicates of soybean seeds in the R6.5 stage, during which there was a dramatic increase in the isoflavone content (Figure 1). A total of 278,070,242 clean reads were generated, with an average length of 151 bp for each sample. The proportion of bases with a Phred quality score of 30 (Q30) ranged from 92.57% to 94.01% (Table 1), with an average of 92.91%, reflecting the high quality of the sequencing data. After mapping the assembled transcripts, 134,591,586 trimmed reads were obtained, with an average of 14,954,621 per sample. These reads were mapped to the reference genome sequence, with an average mapping rate of 96.81%. The mapped reads were then used to calculate normalized read counts to determine gene expression levels. Of the 56,044 reference transcripts (92,028,847 bp long), 39,156 (69.87%) were expressed (Table S2), among which 36,257 were annotated according to Wm82.a2.v1 from the Phytozome database (Table S3). To assess the reproducibility of the results among the three biological replicates for each sample, a hierarchical cluster analysis of gene expression patterns was performed using Pearson correlation coefficients (Figure 2a). The correlation coefficients among the three replicates of Danbaek, DB-088, and Hwanggeum ranged from 0.96 to 0.98, indicative of the high reproducibility of the RNA-seq data.

### 2.3. Analysis of Differential Gene Expression in Soybean Seeds with Distinct Isoflavone Contents

The DEGs between the genotypes with low isoflavone contents (Danbaek and Hwanggeum) and the genotype with a high isoflavone content (DB-088) at the R6.5 stage were analyzed (Figure 2b). A total of 3794 DEGs were detected by the DB-088 vs. Danbaek comparison, including 2314 up-regulated and 1480 down-regulated DEGs. The DB-088 vs. Hwanggeum comparison detected 4405 DEGs, including 2606 up-regulated and 1799 down-regulated DEGs. Among the up-regulated DEGs, 1261 were common to both comparisons, whereas 1053 and 1345 were exclusive to the DB-088 vs. Danbaek and DB-088 vs. Hwanggeum comparisons, respectively. In terms of the down-regulated DEGs, 718 DEGs were common to both comparisons, but 762 and 1081 were detected only in the DB-088 vs. Danbaek and DB-088 vs. Hwanggeum comparisons, respectively (Figure 2c). Of the up-regulated isoflavone-related DEGs, 23 were common to both comparisons, whereas 16 and 12 were exclusive to the DB-088 vs. Danbaek and DB-088 vs. Hwanggeum comparisons, respectively. In terms of the down-regulated isoflavone-related DEGs, 16 were common to both comparisons, but 10 and 28 were detected only in the DB-088 vs. Danbaek and DB-088 vs. Hwanggeum comparisons, respectively (Figure 2d).

**Figure 3:**
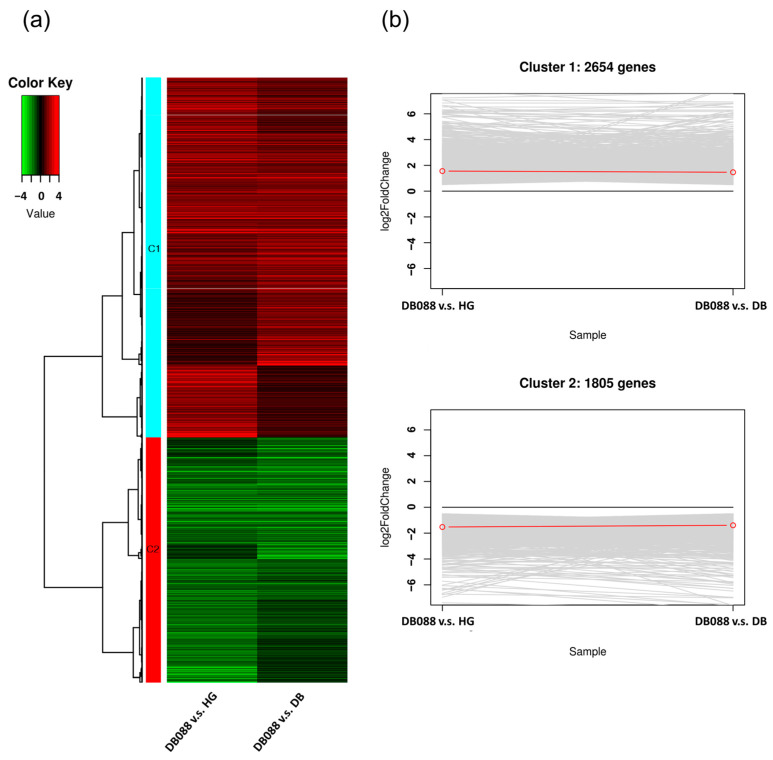
Cluster analysis of common DEGs in Hwanggeum and Danbaek revealed by the comparison with DB-088. (**a**) Heatmap illustrating the expression patterns of the common DEGs in Hwanggeum and Danbaek. C1, cluster 1 representing up-regulated DEGs; C2, cluster 2 representing down-regulated DEGs. (**b**) Line plots depicting the cluster patterns in the heatmap.

Notably, the comparisons with DB-088 revealed 4459 DEGs with similar expression patterns in the genotypes with low isoflavone contents (2654 up-regulated DEGs and 1805 down-regulated DEGs) (Figure 3a,b). The isoflavone biosynthetic genes and the genes encoding the closely related TFs among the DEGs detected by the DB-088 vs. Hwanggeum and DB-088 vs. Danbaek comparisons were verified according to the associated KEGG pathways (Figure S2). Specifically, 23, 12, and 19 DEGs were related to phenylpropanoid, flavonoid, and isoflavone biosynthetic pathways, respectively. In addition, DEGs encoding 94 TFs were identified, including 11 WRKY, 3 MADS-box, 26 MYB, 13 bZIP, 28 WD40, and 13 bHLH TFs. The identification of these DEGs may be important for further clarifying the genes and TFs responsible for isoflavone biosynthesis and accumulation in soybean seeds.

### 2.4. GO and KEGG Enrichment Analyses of DEGs

**Figure 4:**
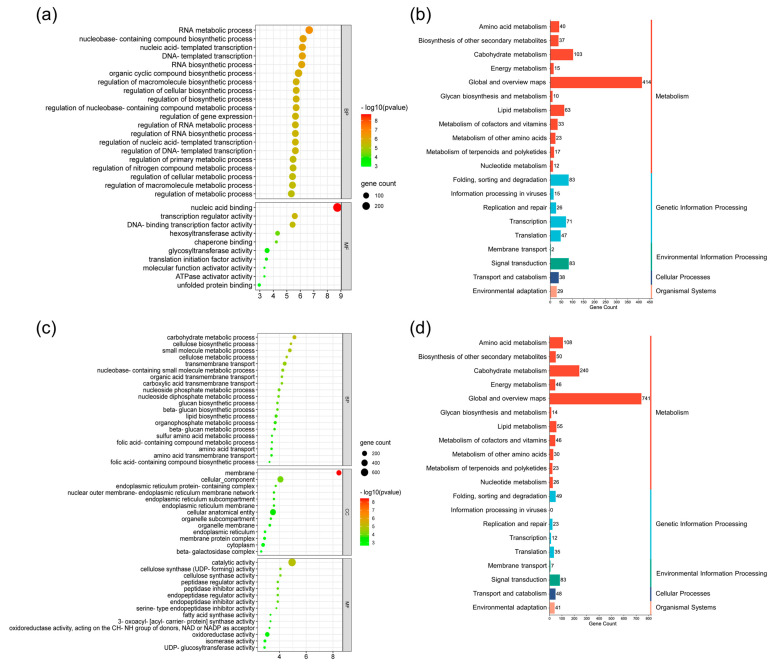
GO and KEGG enrichment analyses of the common DEGs in Hwanggeum and Danbaek revealed by the comparison with DB-088. (**a**,**b**) Enriched GO terms and KEGG categories among the up-regulated DEGs, respectively. (**c**,**d**) Enriched GO terms and KEGG categories among the down-regulated DEGs, respectively.

The GO analysis classified the DEGs into three functional groups (BP, CC, and MF). Respectively, a total of 2654 up-regulated DEGs (cluster 1) and 1805 down-regulated DEGs (cluster 2) were annotated with 55 GO terms (45 and 10 from the BP and MF categories, respectively) and 94 GO terms (67, 13, and 14 from the BP, CC, and MF categories, respectively) (*p* < 0.01). Among the up-regulated DEGs, the most significant BP GO terms were RNA metabolic process (GO:0016070) and nucleobase-containing compound biosynthetic process (GO:0034654) (*p* < 0.001), and the most significant MF GO terms were nucleic acid binding (GO:0003676), transcription regulator activity (GO:0140110), and DNA-binding transcription factor activity (GO:0003700) (*p* < 0.001) (Table S4 and Figure 4a). In terms of the down-regulated DEGs, the three most significant BP GO terms were carbohydrate metabolic process (GO:0005975), cellulose biosynthetic process (GO:0030244), and small-molecule metabolic process (GO:0044281) (*p* < 0.001). The main CC GO terms were membrane (GO:0016020), cellular component (GO:0005575), and cellular anatomical entity (GO:0110165). The major MF GO terms were catalytic activity (GO:0003824) and oxidoreductase activity (GO:0016491) (*p* < 0.001) (Table S5 and Figure 4c).

The common DEGs detected by the comparisons between DB-088 and Danbaek/Hwanggeum were also functionally characterized via a KEGG enrichment analysis (Table S6 and Figure 4b,d). A total of 123 and 122 KEGG pathways were associated with the up-regulated and down-regulated DEGs, respectively. These pathways belonged to the following categories: metabolism (11), genetic information processing (5), environmental information processing (2), cellular processes (1), and organismal systems (1). The largest category was metabolism (1494 up-regulated and 2650 down-regulated DEGs), with 11 sub-categories, such as global and overview maps (414 up-regulated and 741 down-regulated DEGs), which included metabolic pathways (206 up-regulated and 331 down-regulated DEGs) and biosynthesis of secondary metabolites (108 up-regulated and 205 down-regulated DEGs). Genetic information processing (401 up-regulated and 189 down-regulated DEGs) was the second largest category, with five sub-categories, including folding, sorting, and degradation (83 up-regulated and 49 down-regulated DEGs). The third largest category was environmental information processing (85 up-regulated and 90 down-regulated DEGs), with two sub-categories, including signal transduction (83 up-regulated and 83 down-regulated DEGs). The fourth largest category was cellular processes (38 up-regulated and 48 down-regulated DEGs), with endocytosis revealed as a major pathway (20 up-regulated and 15 down-regulated DEGs). Within organismal systems (29 up-regulated and 41 down-regulated DEGs), which was the fifth largest category, plant–pathogen interaction was the main pathway (19 up-regulated and 27 down-regulated DEGs).

### 2.5. Expression Patterns of the Downstream Genes in the Isoflavone Biosynthetic Pathway

**Figure 5:**
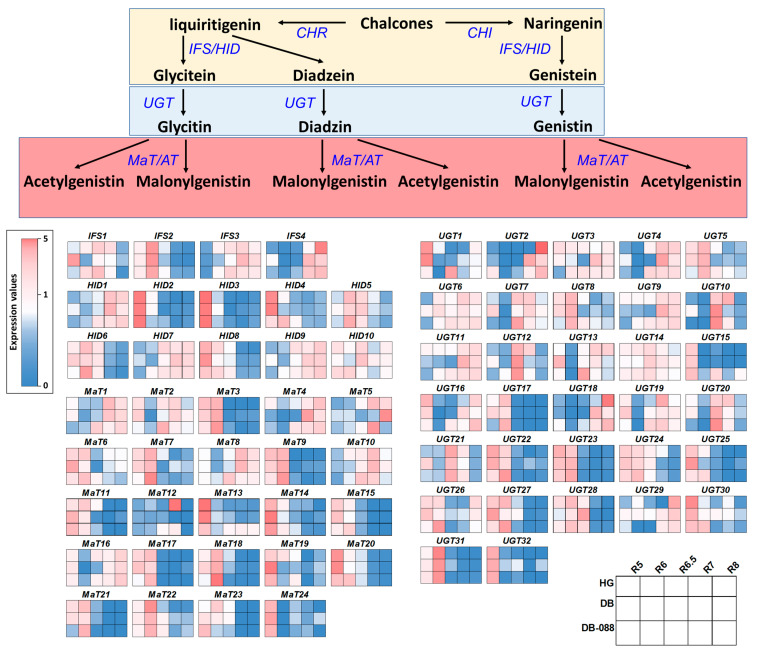
Expression patterns of the downstream genes in the isoflavone biosynthetic pathway among various seed developmental stages on the basis of RNA-seq data and the results of earlier research. The heatmap displays the relative expression levels of the downstream genes in the isoflavone biosynthetic pathway. The expression data were normalized against the expression of the internal reference gene *F-box*.

A qRT-PCR analysis was completed to explore and validate the DEGs involved in the formation of isoflavone glycosides through glycosylation and malonylation. We focused on the downstream genes in the isoflavone biosynthetic pathway, including 32 *UGT*, 24 *MaT*, 10 *HID*, and 4 *IFS* homologs, because of our RNA-seq data and the findings of an earlier study by Hu et al. [37] (Table S7). The downstream genes had similar relative expression patterns in the selected seed developmental stages in all genotypes (Figure 5 and Figure S3). More specifically, *IFS1* and *IFS2* were expressed in the early and middle stages, whereas *IFS3* and *IFS4* were expressed in the late stage. The relative expression levels of the *IFS* genes (excluding *IFS2*) were higher in DB-088 than in Hwanggeum and Danbaek between the R6 and R6.5 stages. Specifically, the *IFS4* (*Glyma.03G143700*) expression level was 3.74- and 7.48-fold higher in DB-088 than in Hwanggeum and Danbaek, respectively. Similarly, the relative expression levels of the *HID* genes (excluding *HID8*) were higher in DB-088 than in Hwanggeum and Danbaek between the R6 and R6.5 stages. The *HID2* (*Glyma.02G134200*) and *HID3* (*Glyma.07G211000*) expression levels were 3.55/10.37-fold and 4.76/9.99-fold higher in DB-088 than in Hwanggeum/Danbaek in the R6.5 stage, respectively. The *UGT* expression patterns varied depending on the developmental stage. However, the relative expression levels of the *UGT* genes increased in DB-088 in the R6 and R6.5 stages. Moreover, the *UGT1* (*Glyma.19G187000*), *UGT2* (*Glyma.11G000500*), *UGT15* (*Glyma.17G019600*), and *UGT16* (*Glyma.02G029900*) expression levels in DB-088 peaked in the R6.5 stage and were 122.19/30.3-fold, 45.68/107.87-fold, 18.69/46.48-fold, and 17.31/26.40-fold higher than the corresponding expression levels in Hwanggeum/Danbaek, respectively. Consistent with the increased expression of *UGT* genes, the isoflavone glycoside contents were 3.35- and 4.91-fold higher in DB-088 than in Hwanggeum and Danbaek, respectively. The expression patterns of the *MaT* genes were similar to those of the *UGT* genes. The relative expression levels of most of the *MaT* genes increased in DB-088 between the R6 and R6.5 stages, including *MaT1* (*Glyma.04G040400*; 7.99- and 6.57-fold), *MaT9* (*Glyma.18G029900*; 42.91- and 4.81-fold), *MaT15* (*Glyma.11G231600*; 2.02- and 3.26-fold), and *MaT20* (*Glyma.16G180500*; 2.74- and 4.08-fold). In contrast, the expression levels of several other *MaT* genes increased from R6.5 to R8, including *MaT3* (*Glyma.11G227600*), *MaT5* (*Glyma.17G152600*), and *MaT13* (*Glyma.03G246600*). Additionally, the malonyl glycoside contents were 3.16/4.91-fold and 3.74/4.48-fold higher in DB-088 than in Hwanggeum/Danbaek in the R6.5 and R7 stages, respectively.

**Figure 6:**
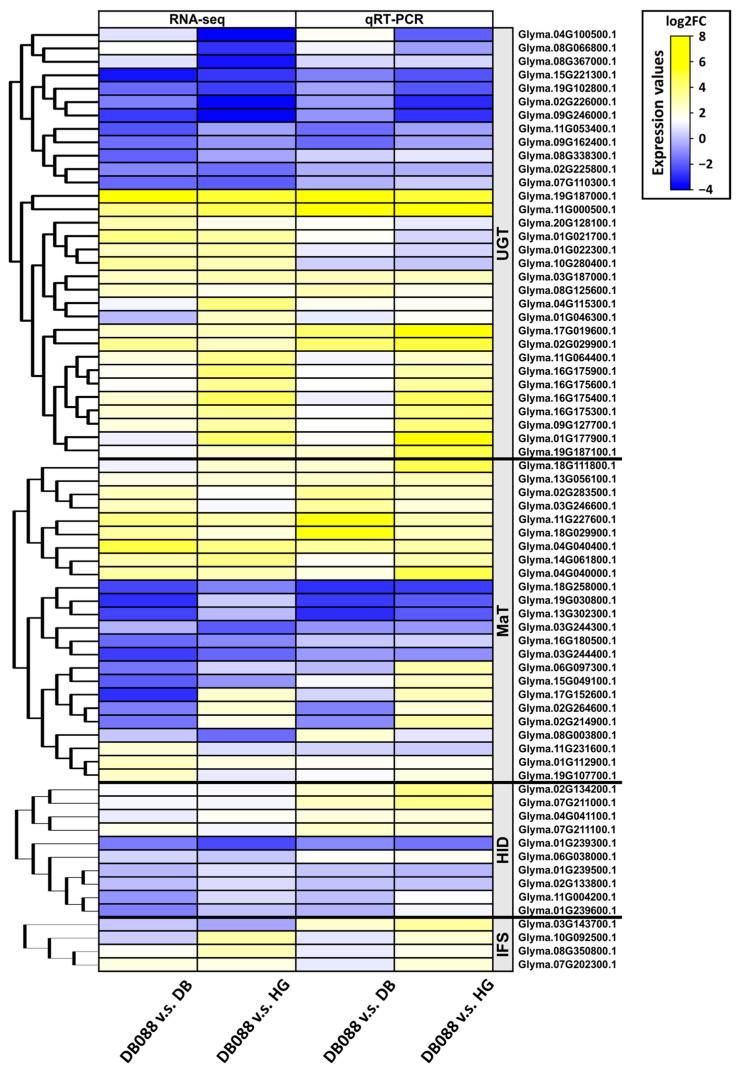
Validation of the relative expression patterns of the downstream genes in the isoflavone biosynthetic pathway in the R6.5 stage (DB-088 vs. Hwanggeum/Danbaek) on the basis of a qRT-PCR analysis. The heatmap illustrates the relative expression levels of the downstream genes in the isoflavone biosynthetic pathway. The expression data were normalized against the expression of the internal reference gene *F-box*.

The analysis of the isoflavone biosynthetic genes detected common expression patterns or expression patterns that were specific to seed developmental stages or the genotypes with low isoflavone contents. In addition, the qRT-PCR data were consistent with the RNA-seq data, indicative of the reliability of the transcriptome results (Figure 6).

### 2.6. Co-Expression Network Analysis of the DEGs Related to Isoflavone Biosynthesis

**Figure 7:**
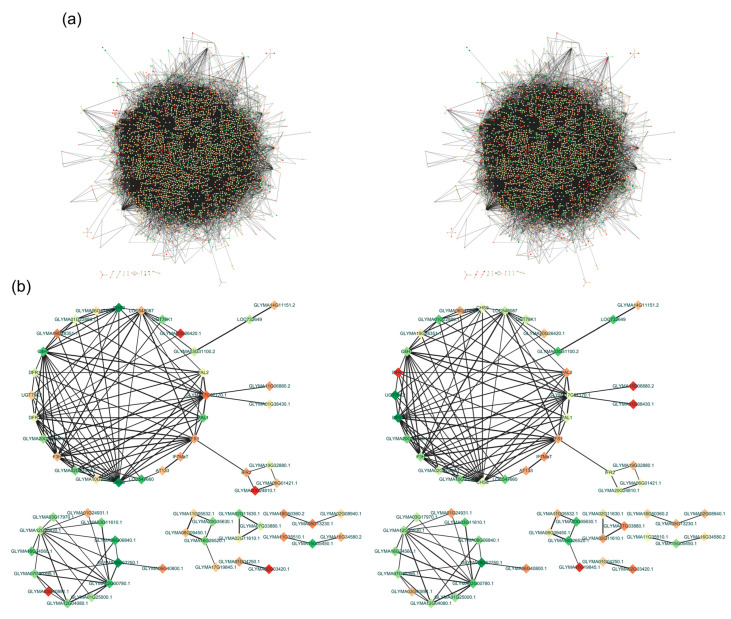
Protein–protein interactions revealed by the analyses of all DEGs and the isoflavone-related DEGs. Edges in the network indicate protein–protein interactions (gray lines). The colored nodes (round circles) reflect gene expression levels. Increases in the thickness and intensity of the lines indicate increases in the confidence scores for the interactions. Up-regulated and down-regulated genes are indicated in red and blue, respectively. (**a**) Network presenting the interactions detected by the analysis of all DEGs. (**b**) Network presenting the interactions revealed by the analysis of the DEGs related to isoflavone biosynthesis. The results for Hwanggeum and Danbaek are presented on the left and right, respectively.

The DEGs were used as queries to screen the Cytoscape STRING database for potential protein–protein interactions. The proteins encoded by 4459 DEGs between the low- and high-isoflavone genotypes were revealed to directly or indirectly interact with one or more proteins on the basis of gene co-expression. Thus, 45,920 potential interactions were detected by the analysis of all DEGs (Figure 7a), with 55 interactions revealed by the analysis of the DEGs related to the isoflavone biosynthetic pathway (Figure 7b). The proteins encoded by six *UGT* genes (*Glyma.02G225800*, *Glyma.02G226000*, *Glyma.04G115300*, *Glyma.11G000500*, *Glyma.11G064400*, and *Glyma.20G128100*) and five *MaT* genes (*Glyma.01G112900*, *Glyma.04G040400*, *Glyma.13G056100*, *Glyma.14G061800*, and *Glyma.19G030500*) were predicted to interact with 35 and 6 proteins, respectively. The proteins encoded by *4CL* (*Glyma.13G323000*), *PAL* (*Glyma.20G180800*), *2HID* (*Glyma.01G239300*), and *IFS* (*Glyma.07G202300*) were predicted to interact with 1–12 proteins. Additionally, the proteins encoded by three *UGT* genes were predicted to interact with more than nine proteins, indicative of their pleiotropic effects (Tables S8 and S9). Some of these genes were differentially expressed between Hwanggeum and Danbaek, suggestive of their cultivar-specific expression patterns.

A total of 137 protein–protein interactions involved proteins encoded by 342 DEGs associated with isoflavone biosynthesis (Tables S10 and S11). Of these interactions, 31 were validated by qRT-PCR. The genes encoding proteins with the most interactions (associations with 12–15 proteins) were *IFS* (*Glyma.07G202300*), *MaT* (*Glyma.11G231600*, *Glyma.13G056100*, *Glyma.15G049100*, *Glyma.19G030500*, and *Glyma.19G107700*), and *UGT* (*Glyma.01G036000*, *Glyma.02G029900*, *Glyma.02G081000*, *Glyma.08G066800*, *Glyma.08G125600*, *Glyma.11G064400*, *Glyma.13G203900*, and *Glyma.20G128100*) genes. Of the downstream genes underlying glycosylation and malonylation, *IFS*, *MaT*, and *UGT* were negatively correlated with the common phenylpropanoid pathway genes, including *PAL*, *C4H*, and *CHS*. Notably, six genes were co-regulated in whole and isoflavone-related protein–protein interactions, including two *UGT* genes (*Glyma.11G064400* and *Glyma.20G128100*) encoding proteins that were predicted to interact with 20 proteins, including isoflavone-related proteins and other proteins contributing to isoflavone biosynthesis.

## 3. Discussion

Mutation breeding methods have been used for crop improvement because they involve the introduction of heritable genetic changes (e.g., SNPs, insertions/deletions, and chromosomal deletions), which can lead to diverse changes to morphological characteristics, agronomic traits, and metabolite contents [38,39]. In terms of radiation breeding, Hajika et al. [40] and Lee et al. [41] generated mutant soybean plants lacking three lipoxygenase isozyme-encoding genes (*Lox1*, *Lox2*, and *Lox3*), which were mutated by insertions/deletions and SNPs introduced by gamma irradiation. Hwang et al. [42] determined that the dwarfism of a fast neutron-irradiated mutant (20% shorter than normal) was due to a deletion in the first exon of *Glyma.15G05831*, which encodes a peroxidase. Kim et al. [30] developed a mutant (Hfa180) with a high stearic acid content, which was the result of a deletion in the exon of *GmSACPD-C* induced by gamma irradiation. Combining recent biotechnological advances with radiation breeding may lead to the establishment of informative genetic resources with desirable traits. For example, we previously generated the soybean mutant DB-088, which has an isoflavone content (average of 8018.56 µg g^−1^) that is significantly higher than that of Danbaek (average of 2111.08 µg g^−1^), via gamma-irradiation [43]. The consistently higher isoflavone content in DB-088 than in the wild-type cultivar (Figure S1) may reflect alterations to the genes responsible for isoflavone biosynthesis and accumulation. Isoflavones, which are valuable phytochemicals in soybeans, are critical for plant defense mechanisms, but they also have health-promoting effects. Hence, optimizing their contents has become a major objective of soybean breeding programs [44]. However, the isoflavone content is a complex trait influenced by multiple genes and their interactions with the environment (G × E). Thus, comprehensively characterizing the mechanisms regulating isoflavone contents is challenging [7,45]. Previous studies showed the soybean seed isoflavone content may vary by more than 2-fold from year to year, even for plants growing in the same geographical locations [8,46]. Notably, the isoflavone contents of Hwanggeum, Danbaek, and DB-088 seeds increased by 2.43-, 2.67-, and 4.84-fold, respectively, during the progression from the R6 stage (full-size green seeds) to the R6.5 stage (full-size greenish-yellow seeds). This substantial change prompted us to focus on the R6.5 stage to elucidate the differences in transcript levels between the low-isoflavone and high-isoflavone genotypes (Figure 1). Kim and Chung [47] also reported that isoflavones rapidly accumulate between the R5 (early) and R7 (onset of physiological maturity) stages.

Earlier research focused primarily on the metabolic engineering of the phenylpropanoid pathway in both non-legume and legume crops. Specifically, in terms of soybean research, studies have been conducted to characterize the genes encoding proteins involved in the initial steps of the phenylpropanoid pathway, including *PAL*, *C4H*, *4CL*, *CHS*, *CHR*, and *CHI* [48]. Genistein accumulation in non-leguminous plants (e.g., tobacco and lettuce) has been modulated via the overexpression and suppression of *IFS* genes. Furthermore, the overexpression of *PAL* genes leads to an increase in the genistein content [49]. The genistein biosynthetic pathway competes with the flavonoid and anthocyanin biosynthetic pathways for the naringenin intermediate within the phenylpropanoid pathway. Moreover, DFR and IFS compete for naringenin, resulting in the production of genistein and flavone, respectively [48]. In addition, CHS, CHR, and CHI catalyze the synthesis of the precursors for isoflavone aglycones before the involvement of IFS and HID. The overexpression of *GmCHI1A* reportedly increases soybean seed daidzein and genistein contents by up to 187.23% and 463.93%, respectively [50]. In the current study, we conducted a preliminary gene expression analysis of 35 structural genes reportedly involved in isoflavone biosynthesis (Table S12). During the R6 stage, the expression levels of several genes were significantly up-regulated in DB-088 (relative to the corresponding expression in Hwanggeum and Danbaek), including *PAL1* (2.57- and 6.68-fold, respectively), *C4H* (1.57- and 3.09-fold, respectively), *4CL* (10.05- and 18.03-fold, respectively), *CHI* (1.36- and 5.37-fold, respectively), and *CHI1B* (2.35- and 1.34-fold, respectively) (Figures S4 and S5). Among the *CHS* genes, the relative expression levels of *CHI1*–*CHI4* increased by up to 2.68-fold in the R6 stage, whereas the relative expression levels of *CHS9* and *CHS11* increased by up to 1.84-fold in the R6.5 stage. Considering CHS, CHR, and CHI function in concert to form the precursors of daidzein and glycitein, these results suggest the relatively high isoflavone content in DB-088 may be related to changes in the expression of the genes associated with the relatively early steps of the isoflavone biosynthetic pathway. Similarly, the expression levels of multiple genes associated with the downstream steps increased in various seed developmental stages. These genes with up-regulated expression levels included *UGT73F2*, *1*, and *7* (1.62- to 3.10-fold), *UGT8*, *9*, and *78K* (1.04- to 12.32-fold), and *MT7-1*, *IMaT1*, and *IMaT3* (2.61- to 5.27-fold), indicative of their substantial contributions to enzyme-mediated glycosylation. In soybean seeds, isoflavone conjugates are stored in central vacuoles, wherein they are stable and soluble because of the glycosylation and malonylation catalyzed by UGTs and MaTs [14]. This is consistent with the fact that more than 80% of the isoflavones in seeds are present as glycosides (daidzin, genistin, and glycitin) and malonyl glycosides [51].

The rapid progress in the development of next-generation sequencing techniques has enabled researchers to generate genome and transcriptome data relatively quickly. For example, RNA-seq has emerged as a crucial technique for profiling target DEGs under particular conditions [52]. In the present study, we performed an RNA-seq analysis to compare two low-isoflavone soybean cultivars (Danbaek and Hwanggeum) with the high-isoflavone mutant DB-088. To examine gene expression trends in developing soybean seeds, we screened for homologous genes related to isoflavone biosynthesis on the basis of our RNA-seq data and published research findings [37]. The expression patterns of a number of isoflavone biosynthesis-related genes varied depending on the genotype and seed maturation stage (Figure 5 and Figure S3), with substantial diversity in the expression patterns of some homologs. Based on these findings, further studies are needed to reveal the sequence variants of causal genes associated with isoflavone biosynthesis and accumulation using whole-genome resequencing.

In this study, *HID* genes were revealed to belong to the alpha/beta-hydrolase superfamily. Notably, increases in *HID7* (*Glyma.11G004200*) and *HID9* (*Glyma.01G239600*) expression are consistent with increases in the coumestan isoflavone content in soybean leaves [53]. The *HID7* gene, which was previously known as *HIDH2* (*Glyma.11G00650*), includes a region that is identical to part of the *HIDH1* (*Glyma.01G45020*) sequence and is predominantly expressed in the pods and shoot [54]. Furthermore, the expression levels of the previously unannotated genes *HID2*, *HID3*, and *HID4* were up to 10-fold higher in DB-088 than in the low-isoflavone cultivars in the R6.5 stage. Of the *IFS* genes, *IFS3* (*Glyma.07G202300*), which was previously known as *IFS1* (encoding isoflavone synthase 1), was identified as a major gene in the isoflavone biosynthetic pathway. In contrast, *IFS2* (*Glyma.08G350800*), which encodes a CYP450 known as CYP93E1, is involved in the production of soya-saponin aglycones and it is co-expressed with ABC transporter genes (*Glyma.15G148500* and *Glyma.19G021500*) [19]. Interestingly, these two *IFS* homologs were co-expressed with ABC and MATE transporter genes, implying the transporter genes and isoflavone biosynthetic genes encode proteins that mediate the vacuolar storage and secretion of various isoflavones and flavonoids [55]. The expression levels of *Glyma.12G177500* and *Glyma.20G123500*, which encode ABC and MATE transporters, were 1.33/1.52-fold and 1.46/3.23-fold higher in DB-088 than in Hwanggeum/Danbaek, respectively.

The expression levels of the *GmIF7GT* homologs, including *UGT3* (*Glyma.16G175400*, *UGT4*), *UGT5* (*Glyma.16G175900*, *UGT7*), *UGT8* (*Glyma.16G175600*, *IF7GT*), and *UGT9* (*Glyma.16G175300*, *UGT2*), were up to 18.41-fold higher in DB-088 than in the low-isoflavone genotypes in the R6.5 stage. These *UGT* homologs correspond to the following previously identified isoflavone 7-*O*-glucosyltransferase genes: *GmIF7GT1* (*UGT1*), *GmIF7GT2* (*UGT2*), and *GmIF7GT4* (*UGT4*). In a previous study, the EST contigs of *GmIF7GT1* and *GmIF7GT5* were more than 99% similar to sequences on chromosome 16 [56]. Both *GmIF7GT1* and *GmIF7GT4* are highly expressed in cotyledons, whereas *GmIF7GT2* is abundantly expressed specifically in the roots [54]. Thus, *UGT* homologs encode enzymes with specificity for substrates related to isoflavones and flavonoids in various soybean tissues, including leaves, roots, seeds, and stems [57]. Moreover, the expression levels of *UGT1* (*Glyma.19G187000*) and *UGT2* (*Glyma.11G000500*), which encode glycosyltransferases, were significantly up-regulated in DB-088. More specifically, the *UGT1* and *UGT2* expression levels in the R6.5 stage were 122.19/30.30-fold and 45.68/107.87-fold higher in DB-088 than in Hwanggeum/Danbaek, respectively. Yin et al. [58] reported that six UGTs catalyze the conversion of flavonol, isoflavone, flavone, and flavanol aglycones to glycosides and glycoconjugates. Notably, the enzyme encoded by *UGT732C20* (*Glyma.19G187000*) is highly active when flavonol and isoflavone aglycones are used as substrates. The overexpression of *UGT1* leads to increases in isoflavone glycoside and flavonol contents in soybean.

The BAHD family of acyltransferases (acetyl-CoA, malonyl-CoA, and coumaroyl-CoA) is crucial for the formation of isoflavone glycosides. These enzymes participate in the modification, transport, and subsequent storage of malonylated isoflavones within vacuoles. In the current study, *MaT21* (*Glyma.18G258000*) and *MaT8* (*Glyma.13G056100*) were identified as *GmIMaT2* and *GmIMaT3*, respectively [17,18]. Both of these *MaT* genes were expressed at higher levels in DB-088 than in the low-isoflavone cultivars. Additionally, the isoflavone 7-*O*-glucoside-6″-*O*-malonyltransferase gene *MaT17* (*Glyma.19G030800*), which is located adjacent to qDAID03 and qGLY04, is highly expressed specifically in genotypes with high isoflavone contents [59]. A recent study showed that *MaT18* (*Glyma.13G302300*), which is similar to *GmMaT2*, is highly expressed in seeds and pods [18]. The functional characterization of MtMaT1, 2, and 3 indicated that they perceive a range of glycosides and facilitate the accumulation of malonylated isoflavones [15]. Similarly, MtMaT4, 5, and 6 convert glycosides to malonyl glycosides in *Medicago truncatula* [22]. In soybeans, UGT73F2 exhibits specificity for aglycones, including genistein and glycitein. Recombinant GmMT7 displays high specificity for glycosides in soybean seeds [14]. The specificity of GmIMaT1 and GmIMaT3 for isoflavone 7-*O*-glucosides as substrates has been demonstrated. The overexpression and knockdown of the corresponding genes lead to increases and decreases in malonyl glycoside production, respectively [17]. Similarly, recombinant GmMaT2 and GmMaT4 mediate the transformation of isoflavone 7-*O*-glucosides to malonylated compounds. Overexpressing *GmMaT2* and *GmMaT4* in transgenic hairy roots promotes nodule formation and the production of malonyl glycosides [18]. Although some *MaT* genes, such as *MaT1*–*MaT6* (*Glyma.04G040400*, *Glyma.14G061800*, *Glyma.11G227600*, *Glyma.04G40000*, *Glyma.17G152600*, and *Glyma.18G111800*) and *MaT13* (*Glyma.03G246600*), have not been previously characterized, we observed that they were highly expressed in DB-088 (up-regulated by up to 60-fold). Accordingly, the encoded proteins may substantially contribute to the formation of isoflavone conjugates. As a result of the multiple alignment of these isoflavone biosynthetic genes in soybean, including IFS, HID, UGT, MaT, it is known to regulate the glycosylation and malonylation of glycoconjugates from *Glycine soja* and *Medicago sativa* (Figure S6a–d).

The production of secondary metabolites, including phenolic compounds, is regulated by biosynthetic genes as well as by TFs, such as bHLH, WRKY, and MYB TFs, in numerous higher plants [60,61]. In this study, notable changes in the expression of 81 TF genes were detected in the three genotypes with varying isoflavone contents (Figure S2). The WRKY TFs help protect plants from biotic stresses (e.g., pathogen-induced diseases) and abiotic stresses (e.g., environmental stimuli), while also participating in the signaling pathways of plant hormones, including abscisic acid in soybean [62,63]. They can also collaborate with MYB TFs to modulate responses to abiotic and biotic stresses [64]. In the MADS-box family, *Glyma.08G105500*, which encodes SEPALLATA3, has regulatory effects on the soybean floral meristem (flowering and maturation) [65]. The expression of this gene was up-regulated in DB-088. The MADS-box TFs have important roles related to the development of floral organs as well as the biosynthesis of anthocyanins [66]. Among the MYB TF family members, *Glyma.07G054000* and *Glyma.16G023000* (*MYB111*) had significantly up-regulated expression levels (approximately 3-fold) in DB-088. Earlier research indicated MYB111, which is a member of the R2R3-MYB family, specifically targets genes associated with flavonoid biosynthesis (*CHS*, *CHI*, and *UGT*) in cotyledons [67]. Chu et al. [25] reported that the R2R3-type MYB TF GmMYB29 activates the expression of isoflavone biosynthetic genes, including *IFS2* and *CHS8*. The overexpression and silencing of *GmMYB29* increase and decrease the isoflavone content in soybean hairy roots, respectively. In terms of the bZIP TF genes, *Glyma.04G026200* (*bZIP31*) was more highly expressed in DB-088 than in the low-isoflavone genotypes (1.36- and 1.53-fold higher). On the basis of the results of an earlier study involving gene overexpression and RNAi-based silencing, the bZIP TF GmbZIP5, which can interact with GmMYB176, helps control glyceollin, isowighteone (phytoalexin), and isoflavone contents [68]. Metabolic regulatory mechanisms involving the MYB–bHLH–WD40 complex affect the construction and transformation of isoflavone precursors. The functional annotation and analysis of the genotypes with high and low isoflavone contents using Cytoscape STRING revealed the co-regulation of various proteins associated with isoflavone biosynthesis. The *IFS* (*Glyma.07g202300*), *UGT* (*Glyma.11g064400* and *Glyma.20g128100*), and *MaT* (*Glyma.13g056100* and *Glyma.19g030500*) genes were revealed to be co-regulated, suggestive of their substantial roles in isoflavone biosynthesis and accumulation in soybean seeds.

## 4. Materials and Methods

### 4.1. Plant Materials

The soybean mutant DB-088 was created by treating the Korean soybean cultivar Danbaek with 250 Gy ^60^Co gamma rays at the Korea Atomic Energy Research Institute (KAERI; Jeongeup, 35.51° N 126.83° E, Republic of Korea) [43]. Korean soybean cultivars Hwanggeum and Danbaek (low isoflavone contents) and DB-088 (high isoflavone content) were included in the RNA-seq analysis performed to examine the key genes related to isoflavone biosynthesis. Ten plants per genotype were grown in 20 cm pots filled with commercial soil (Hungnong, Pyeongtaek, Republic of Korea) in the Radiation Breeding Research Greenhouse at KAERI.

### 4.2. Determination of Isoflavone Contents

Isoflavones were extracted from soybean seeds at different developmental stages, including the reproductive stages R5, R6, R6.5, R7, and R8, with three replicates per stage as described by Kim et al. [35]. The seeds were dried and ground to a fine powder using the Geno/Grinder tissue homogenizer (SPEX SamplePrep LLC., Metuchen, NJ, USA), after which 20 mg powdered material was transferred to 2 mL microcentrifuge tubes containing 1 mL 80% (*v*/*v*) methanol. The filtered extracts were subjected to an ultra-performance liquid chromatography (UPLC) analysis. The UPLC system was equipped with a reversed-phase UPLC column (ACQUITY BEH C18 column; 2.1 × 50 mm, 1.7 µm particle size; Waters Corp., Milford, MA, USA). The following 12 isoflavone standards were purchased from Sigma-Aldrich (St. Louis, MO, USA) for the identification and quantification of individual isoflavones and the analysis of the TI contents in soybean seeds: daidzein, glycitein, genistein, daidzin, glycitin, genistin, malonyl daidzin, malonyl glycitin, malonyl genistin, acetyl daidzin, acetyl glycitin, and acetyl genistin.

### 4.3. RNA Extraction

Total RNA was isolated from three replicates of Hwanggeum, Danbaek, and DB-088 seeds at the R6.5 stage. All seed samples were immediately frozen in liquid nitrogen and then stored in a freezer until they were analyzed. Frozen seeds (100 mg) were ground to a fine powder. Fruit-mate (Takara, Shiga, Japan) and the TRIzol reagent (Invitrogen, Carlsbad, CA, USA) were used to purify RNA. More specifically, 100 mg ground seed material was pretreated with 1 mL Fruit-mate reagent to remove polysaccharides/polyphenols. The supernatant (800 µL) was mixed with 800 µL TRIzol reagent and then the solution was incubated for 5 min to allow for complete dissociation. After adding 200 µL chloroform to lyse the cells, the mixture was shaken thoroughly. Next, 800 µL isopropanol was added to the aqueous phase and the mixture was incubated for 40 min at −20 °C to precipitate the RNA. The RNA pellet was washed with 75% ethanol and then resuspended in DEPC-treated water. The purity of the extracted total RNA was assessed using the NanoDrop ND-1000 spectrophotometer (NanoDrop Technologies, Wilmington, DE, USA).

### 4.4. cDNA Library Construction and RNA Sequencing

To examine the transcripts associated with isoflavone biosynthesis and accumulation, an RNA-seq analysis was conducted using three genotypes with consistently low or high isoflavone contents across 3 years (Figure S1). Paired-end libraries were constructed using the TruSeq^®^ Sample Preparation Kit (Illumina, San Diego, CA, USA). The quality and concentration of each sample were determined using the 2100 Bioanalyzer (Agilent Technologies, Santa Clara, CA, USA). All libraries were sequenced using the Illumina HiSeq X Ten system (Illumina) to generate 150 bp paired-end reads. Briefly, mRNA was purified using poly-T oligo-attached magnetic beads and then chemically fragmented before being converted to single-stranded cDNA using random hexamer primers. Next, the second strand was produced to create double-stranded cDNA. After generating blunt-ended cDNA fragments, an A-tail was added to the blunt ends for the subsequent ligation of sequencing adapters. The cDNA fragments containing adapters were amplified by PCR using adapter-specific primers. All libraries were included in the high-throughput sequencing analysis, which was completed using the Illumina HiSeq X platform. The raw read data (for three replicates of the three genotypes) were deposited in the NCBI Sequence Read Archive under the following accession number: PRJNA1012584.

### 4.5. Data Preprocessing and Classification of Differentially Expressed Genes

The adapter sequence was removed from the transcriptome short reads using Cutadapt. Sequence data with Q ≥ 20 were extracted using DynamicTrim and LengthSort from the SolexaQA package. Trimming resulted in reads with a mean length of 101 bp across all samples and a minimum length of 25 bp. The clean reads were mapped to the reference genome using HISAT2. Gene expression levels were determined according to the total number of reads mapped to each gene using HTSeq (v.0.11.0). The read counts were normalized against the total read count to calculate the relative read counts. The data normalization step was completed using DESeq in the R package. Genes were functionally annotated using the Phytozome database. Available online: https://phytozome.jgi.doe.gov/pz/portal.html (accessed on 1 January 2024).

Significant differentially expressed genes (DEGs) were detected on the basis of a binomial test, with a log_2_(fold-change) ≥ 2 between samples and an adjusted *p*-value (false discovery rate) ≤ 0.01 set as the thresholds for significance. Additionally, log_2_(fold-change) > 1 and log_2_(fold-change) < −1 were used as the thresholds for determining up-regulated and down-regulated gene expression, respectively.

### 4.6. Gene Ontology and Kyoto Encyclopedia of Genes and Genomes Enrichment Analyses and Protein–Protein Interaction Network Analysis

The filtered DEGs were functionally annotated on the basis of Gene Ontology (GO) and Kyoto Encyclopedia of Genes and Genomes (KEGG) enrichment analyses. The GO analysis was performed to divide the filtered DEGs into the molecular function (MF), cellular component (CC), and biological process (BP) categories using the *Glycine max* (Wm82.a2.v1) reference genome (*p* < 0.05) and the agriGO online tool (http://systemsbiology.cpolar.cn/agriGOv2/). The KEGG pathway enrichment analysis was completed using the amino acid sequences in the KEGG database (https://www.genome.jp/kegg) and BLASTP (e-value ≤ 0.01, best hits). The protein–protein interaction network analysis was conducted using StringApp (v.2.0.1). A confidence cutoff value was employed to streamline the networks and remove redundancies. Gene identifiers and descriptions were retrieved from the STRING database (http://string-db.org). The obtained data were analyzed and visualized using Cytoscape 3.9.1 (http://cytoscape.org). All URLs are available online (accessed on 1 January 2024). 

### 4.7. Quantitative Real-Time PCR Analysis

To validate the candidate genes, seeds were collected at five developmental stages (R5, R6, R6.5, R7, and R8). After removing the pod, the samples were immediately frozen in liquid nitrogen. Total RNA was extracted as described above. To remove DNA contaminants, 20 µg total RNA was digested using the DNA-free Kit (Invitrogen, Grand Island, NY, USA). First-strand cDNA was synthesized using the SuperScript III First-Strand Synthesis SuperMix kit (Invitrogen, Carlsbad, CA, USA). The transcript levels of the DEGs were determined by performing a quantitative real-time PCR (qRT-PCR) analysis using the iTaq Universal SYBR Green Supermix (Bio-Rad, Hercules, CA, USA) and the CFX96 Real-Time PCR system (Bio-Rad), with *F-box* serving as the reference gene. The PCR program was as follows: 95 °C for 10 min; 40 cycles of 95 °C for 15 s, 50 °C for 15 s, and 72 °C for 30 s. All samples were analyzed using five replicates. Relative transcript levels were calculated according to a 2^−ΔΔCt^ method. The Primer3Plus program was used to design gene-specific primers according to the coding sequences in SoyBase. Details regarding the primers are provided in Supplementary Tables S6 and S11.

## 5. Conclusions

In this study, we used a high-isoflavone soybean mutant (DB-088) to conduct an in-depth transcriptome analysis, the results of which provide relevant insights into the crucial genes and TFs responsible for the variations in soybean seed isoflavone contents. The identified DEGs encode enzymes that directly affect isoflavone biosynthesis by catalyzing dynamic processes, including glycosylation (UGT) and malonylation (MaT). The study findings have further clarified the molecular basis of isoflavone biosynthesis, while also providing soybean researchers and breeders with valuable genetic information that may be applied to generate soybean varieties with optimal levels of health-promoting isoflavones.

## Figures and Tables

**Table 1:** Information on RNA-seq data in soybean seeds at R6.5 stage.

Sample ID	Raw Reads	Avg. Length (bp)	Total Length (bp)	GC (%)	Q30 (%)	Clean Reads	Mapping Rate	
							NO. Reads	Percent (%)
Danbaek_1	33,164,566	151	5,007,849,466	45.03	94.01	31,428,746	14,831,715	94%
Danbaek_2	33,036,170	151	4,988,461,670	46.73	92.70	30,724,592	15,034,644	98%
Danbaek_3	32,927,962	151	4,972,122,262	46.1	92.74	30,650,778	15,029,361	98%
DB088_1	33,359,878	151	5,037,341,578	46.79	92.93	31,149,264	14,982,713	96%
DB088_2	32,669,030	151	4,933,023,530	47.20	92.69	30,444,128	14,799,324	97%
DB088_3	33,099,718	151	4,998,057,418	46.62	92.57	30,661,986	14,957,058	98%
Hwanggeum_2	33,033,674	151	4,988,084,774	46.99	92.95	30,862,092	14,801,354	96%
Hwanggeum_3	33,363,984	151	5,037,961,584	46.35	92.90	31,142,916	15,097,658	97%
Hwanggeum_5	33,329,700	151	5,032,784,700	46.74	92.67	31,005,740	15,057,759	97%
Total	297,984,682		44,995,686,982			278,070,242	134,591,586	

GC (%): GC content. Q30 (%): ratio of bases that have Phred quality score of over 30. No. Reads: Number of mapped reads.

## Data Availability

The authors will make the dataset from this study available upon receiving reasonable request.

## Supplementary Materials

The following supporting information can be downloaded at https://www.mdpi.com/article/10.3390/plants13050584/s1, Figure S1: Variations in the total isoflavone contents in soybean seeds across 3 years. DB, Danbaek; HG, Hwanggeum; Figure S2: Heatmap depicting the expression patterns of the DEGs related to isoflavone biosynthesis and TFs according to KEGG pathways. Gene expression levels are presented as log_2_(fold-change) values; Figure S3: Relative expression patterns of the downstream genes involved in isoflavone biosynthesis in different seed developmental stages according to the RNA-seq data and the findings of earlier research. The red and blue lines represent the results of the DB-088 vs. Hwanggeum and DB-088 vs. Danbaek comparisons, respectively. The heatmap presents relative gene expression levels, which were normalized against the expression of the internal reference gene *F-box*; Figure S4: Overview of the expression patterns of previously reported genes involved in isoflavone biosynthesis across seed developmental stages. The heatmap presents relative gene expression levels, which were normalized to the expression of the internal reference gene *F-box*; Figure S5: Relative expression patterns of previously reported genes involved in isoflavone biosynthesis across seed developmental stages. The results of the comparisons between DB-088 and Hwanggeum (red line) and Danbaek (blue line) are presented. The heatmap presents relative gene expression levels, which were normalized against the expression of the internal reference gene *F-box*; Figure S6: Phylogenetic analysis based on the homologous amino acid sequences of genes downstream in the isoflavone biosynthetic pathway. (a) UDP-glycosyltransferases. (b) Malonyl-CoA:flavonoid acyltransferases. (c) Isoflavone synthase. (d) 2-hydroxyisoflavanone dehydratase. The analysis was conducted using neighbor-joining method in MEGA X software, with bootstrap values at each branch point derived from 500 replications to assess the branch reliability; Table S1: Individual and total isoflavone contents in different seed developmental stages; Table S2: Information regarding the levels of differentially expressed genes; Table S3: Summary of the reference gene set; Table S4: Information regarding the GO terms in C1; Table S5: Information regarding the GO terms in C2; Table S6: Information regarding KEGG enrichment; Table S7: Information regarding the qRT-PCR primers designed for isoflavone biosynthetic genes; Table S8: Information regarding the STRING network nodes for all DEGs; Table S9: Information regarding the STRING network edges for all DEGs; Table S10: Information regarding the STRING network nodes for isoflavone-related DEGs; Table S11: Information regarding the STRING network edges for isoflavone-related DEGs; Table S12: Information regarding the primers designed for isoflavone biosynthetic genes on the basis of previous studies.
