# Supplementary material for: Differentially Expressed Genes Related to Isoflavone Biosynthesis in a Soybean Mutant Revealed by a Comparative Transcriptomic Analysis
Source: Plants (Basel). 2024 Feb 21;13(5):584. doi: 10.3390/plants13050584 (PMC10934856; doi:10.3390/plants13050584)
Supplement: Supplementary file 1 [file plants-13-00584-s001.zip › Supplementary Figures.pdf]

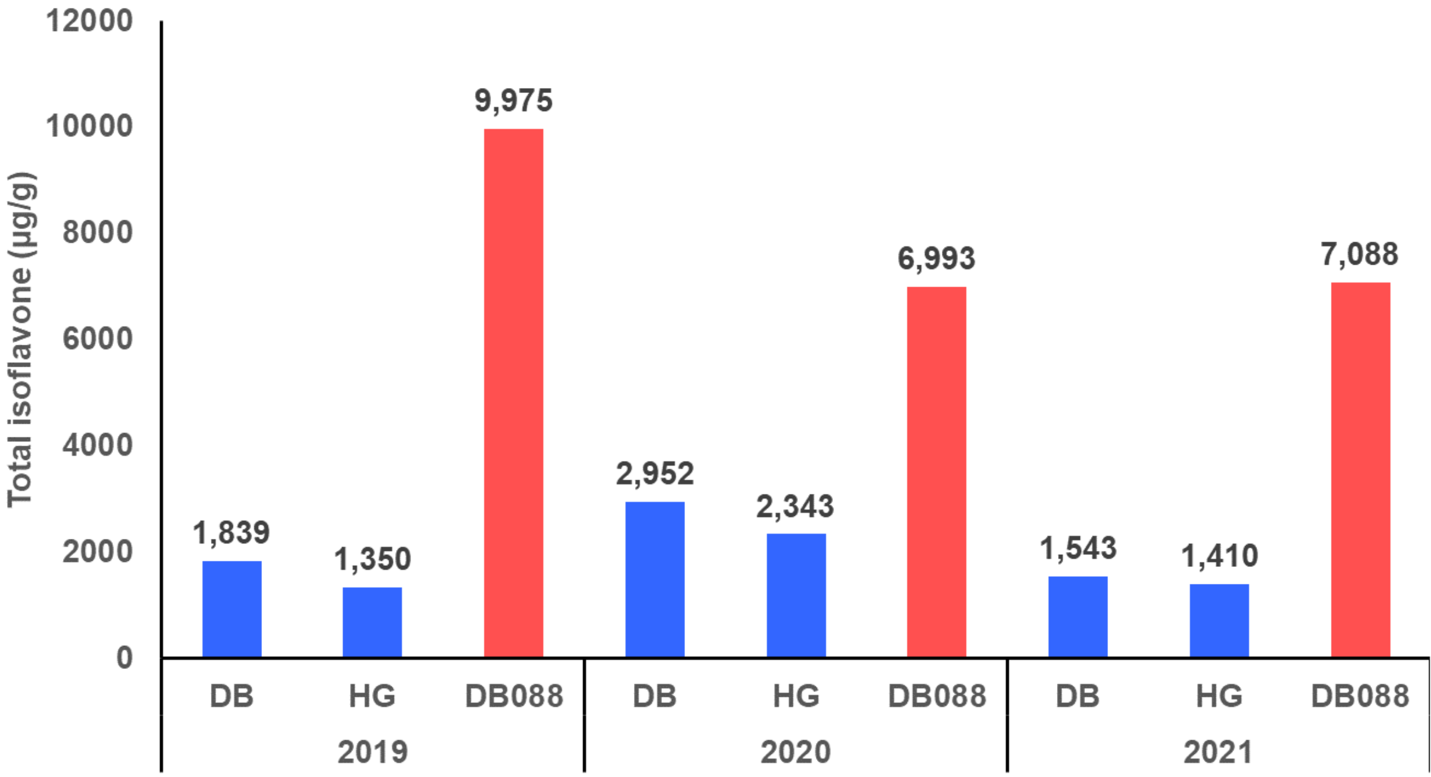

Figure S1. Variations in the total isoflavone contents in soybean seeds across 3 years. DB, Danbaek; HG, Hwanggeum.

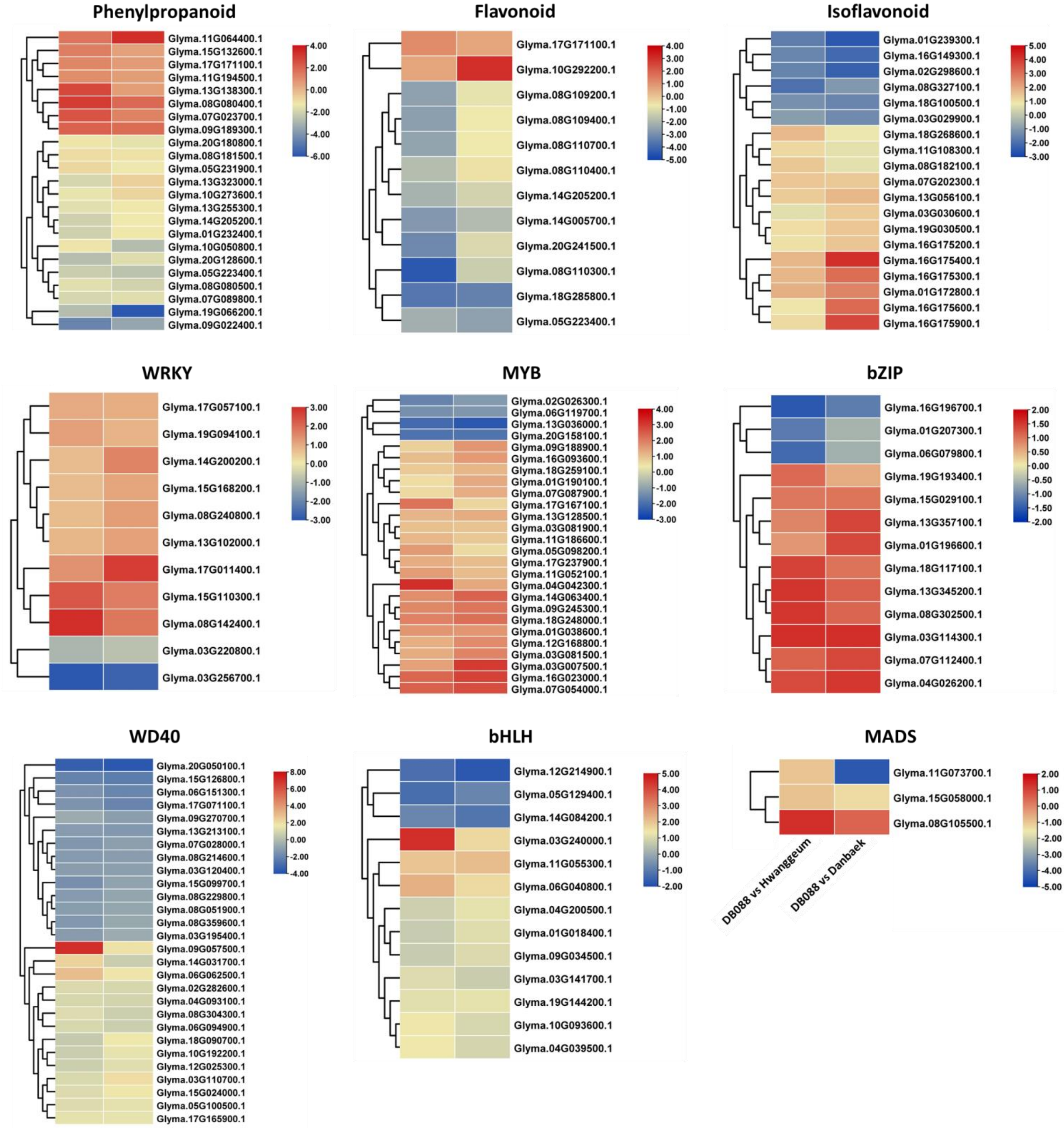

Figure S2. Heatmap depicting the expression patterns of the DEGs related to isoflavone biosynthesis and TFs according to KEGG pathways. Gene expression levels are presented as log_2(fold-change) values. The results for Hwanggeum and Danbaek are presented on the left and right, respectively.

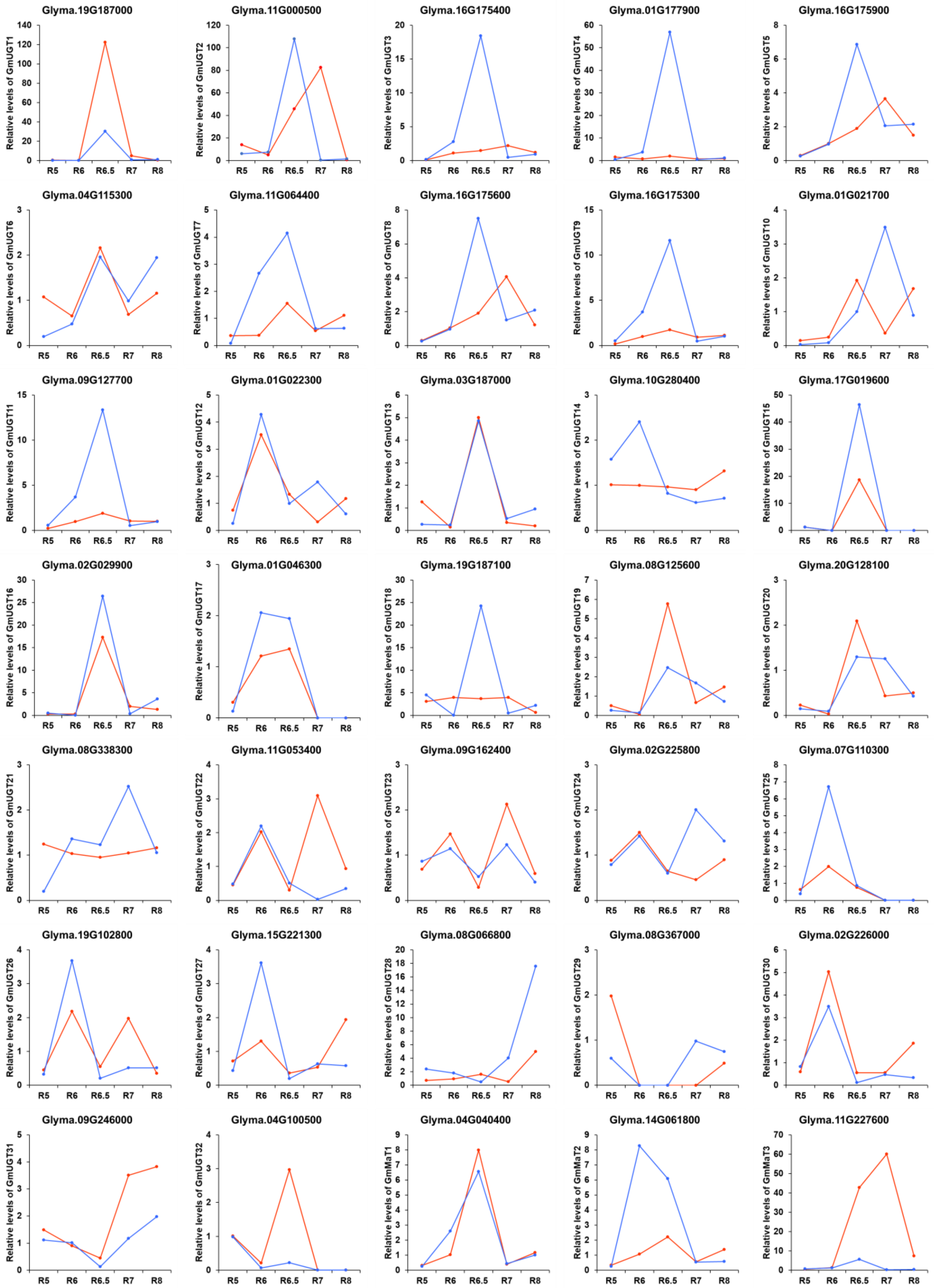

Figure S3. Relative expression patterns of the downstream genes involved in isoflavone biosynthesis in different seed developmental stages according to the RNA-seq data and the findings of earlier research. The red and blue lines represent the results of the DB-088 vs Hwanggeum and DB-088 vs Danbaek comparisons, respectively. The heatmap presents relative gene expression levels, which were normalized against the expression of the internal reference gene *F-box*.

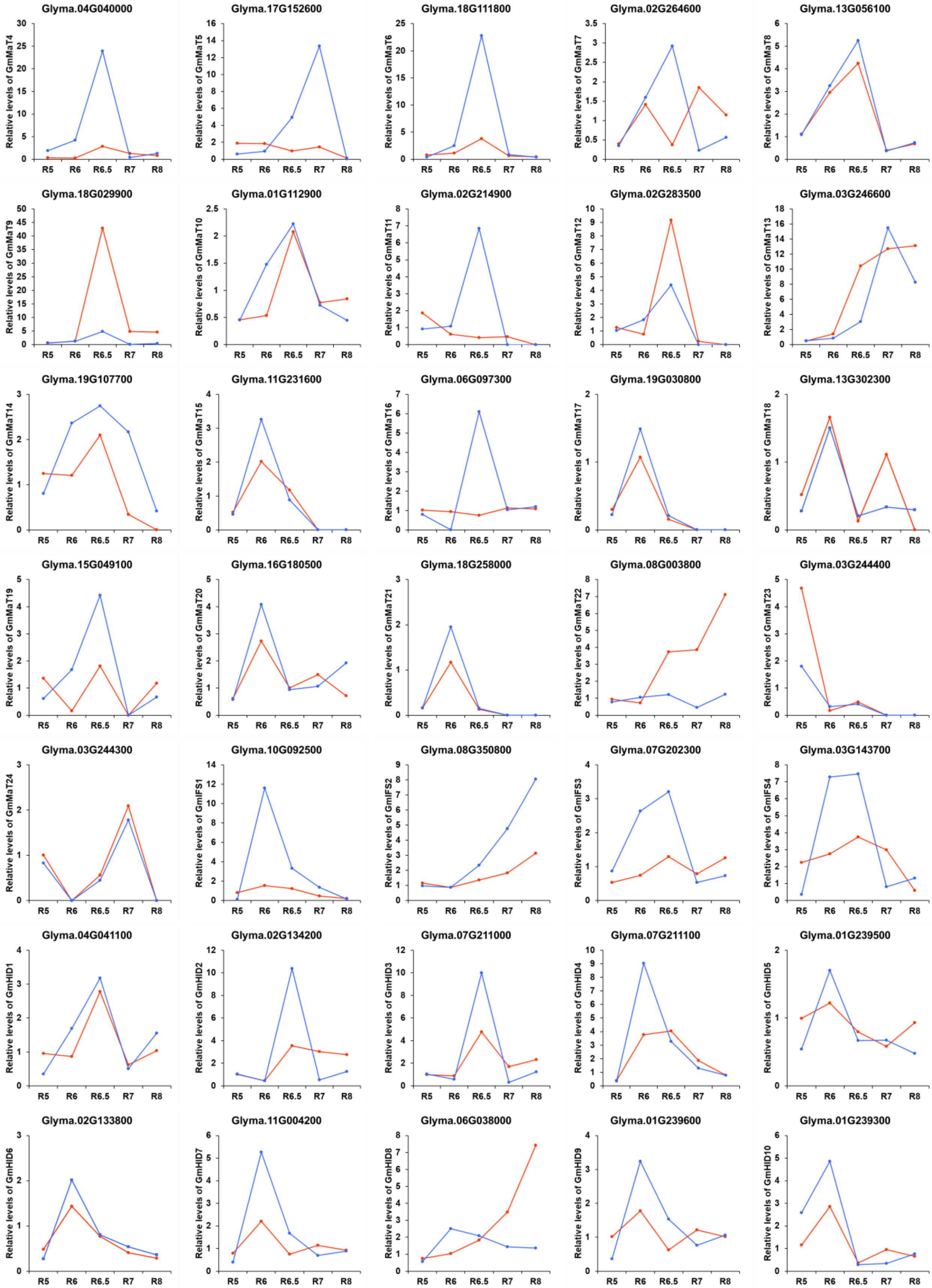

Figure S3. continued.

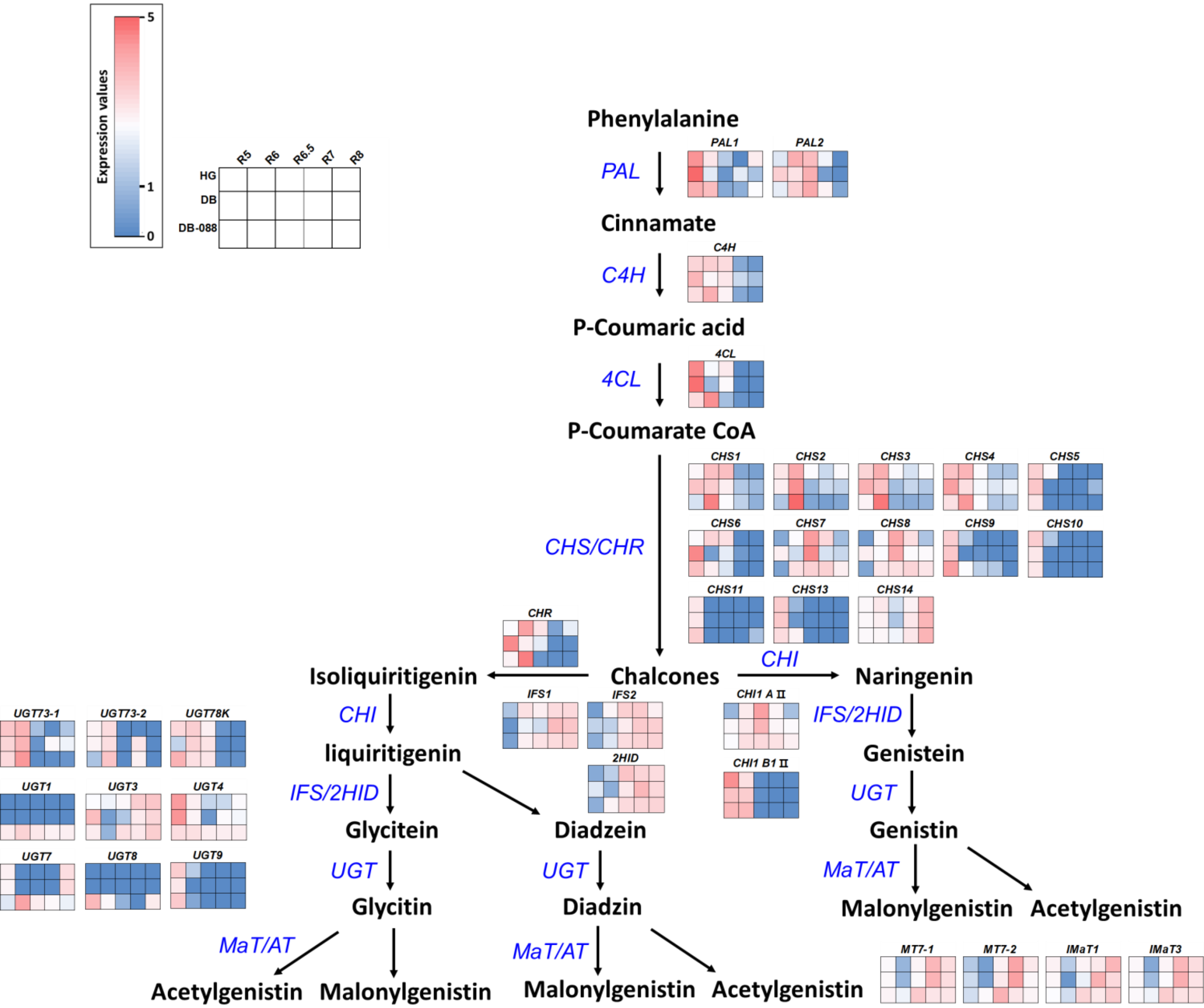

Figure S4. Overview of the expression patterns of previously reported genes involved in isoflavone biosynthesis across seed developmental stages. The heatmap presents relative gene expression levels, which were normalized against the expression of the internal reference gene *F-box*.

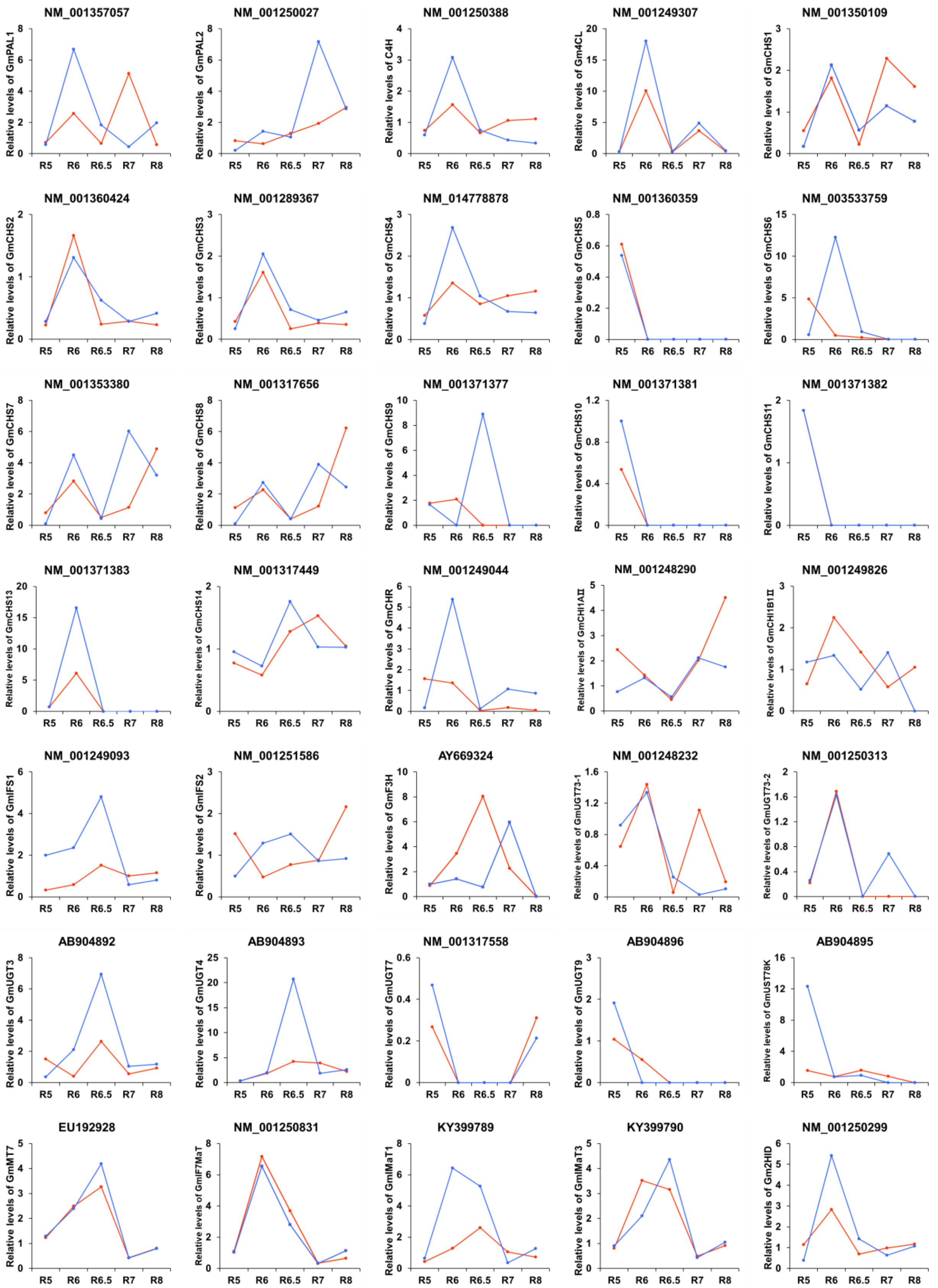

Figure S5. Relative expression patterns of previously reported genes involved in isoflavone biosynthesis across seed developmental stages. The results of the comparisons between DB-088 and Hwanggeum (red line) and Danbaek (blue line) are presented. The heatmap presents relative gene expression levels, which were normalized against the expression of the internal reference gene *F-box*.

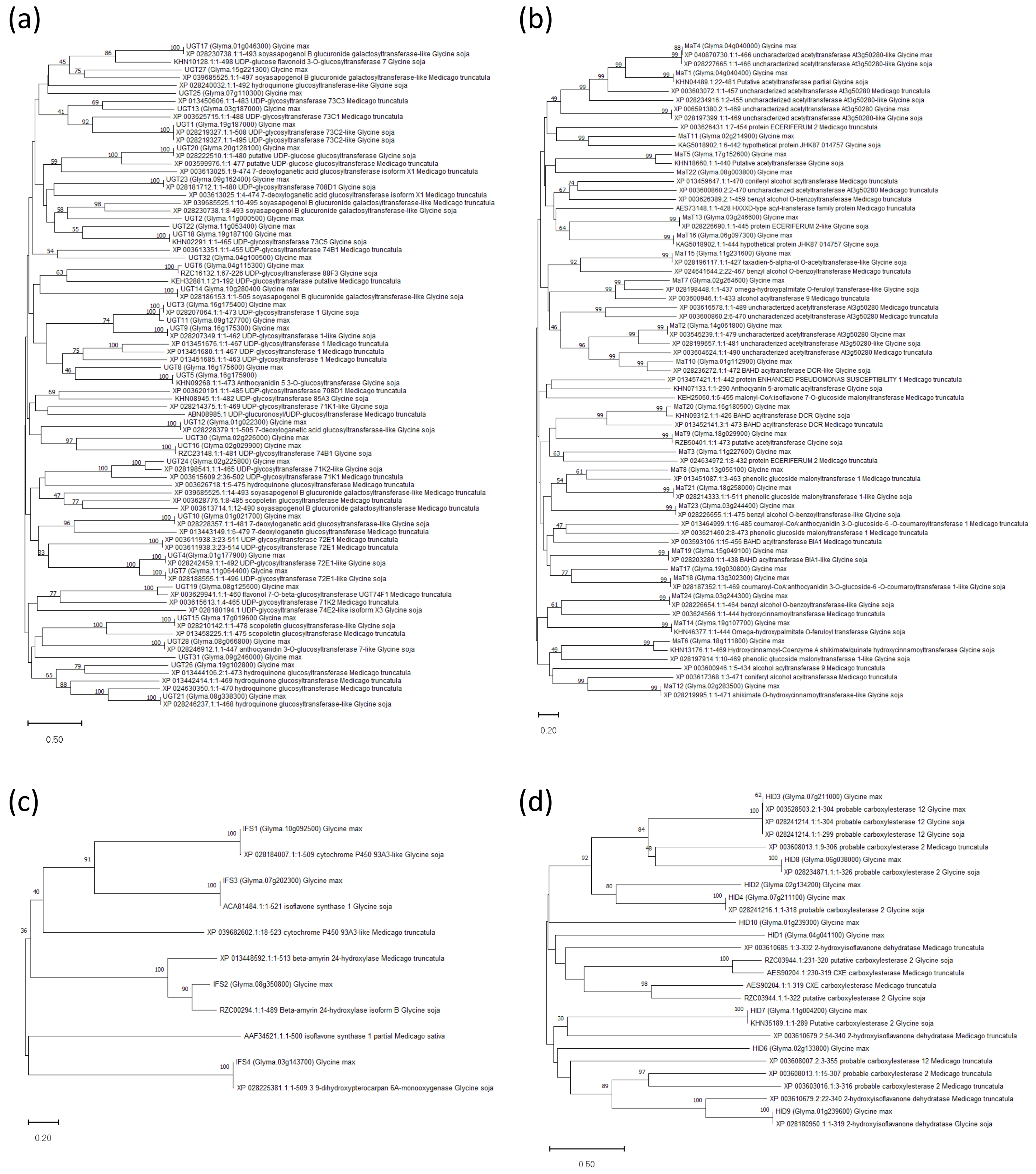

Figure S6. Phylogenetic analysis based on the homologous amino acid sequences of genes downstream in the isoflavone biosynthetic pathway. (a) UDP-glycosyltransferases (UGT). (b) Malonyl-CoA:flavonoid acyltransferases (MaT). (c) Isoflavone synthase (IFS). (d) 2-hydroxyisoflavanone dehydratase (HID). The analysis was conducted using neighbor-joining method in MEGA X software, with bootstrap values at each branch point derived from 500 replications to assess the branch reliability.
